# Prediction models for development of retinopathy in people with type 2 diabetes: systematic review and external validation in a Dutch primary care setting

**DOI:** 10.1007/s00125-020-05134-3

**Published:** 2020-04-03

**Authors:** Amber A. van der Heijden, Giel Nijpels, Fariza Badloe, Heidi L. Lovejoy, Linda M. Peelen, Talitha L. Feenstra, Karel G. M. Moons, Roderick C. Slieker, Ron M. C. Herings, Petra J. M. Elders, Joline W. Beulens

**Affiliations:** 1 Department of General Practice and Elderly Care Medicine, Amsterdam Public Health Research Institute, Amsterdam UMC – location VUmc, van der Boechorststraat 7, 1081 BT Amsterdam, the Netherlands; 2 Julius Center for Health Sciences and Primary Care, University Medical Center Utrecht, Utrecht University, Utrecht, the Netherlands; 3 Groningen Research Institute of Pharmacy, University of Groningen, Groningen, the Netherlands; 4 Centre for Nutrition, Prevention and Health Services, Institute for Public Health and the Environment, Bilthoven, the Netherlands; 5 Department of Epidemiology and Biostatistics, Amsterdam Public Health Research Institute, Amsterdam UMC – location VUmc, Amsterdam, the Netherlands; 6 Department of Cell and Chemical Biology, Leiden University Medical Center, Leiden, the Netherlands; 7 PHARMO Institute for Drug Outcomes Research, Utrecht, the Netherlands

**Keywords:** External validation, Prediction models, Retinal screening, Retinopathy, Systematic review, Type 2 diabetes

## Abstract

**Aims/hypothesis:**

The aims of this study were to identify all published prognostic models predicting retinopathy risk applicable to people with type 2 diabetes, to assess their quality and accuracy, and to validate their predictive accuracy in a head-to-head comparison using an independent type 2 diabetes cohort.

**Methods:**

A systematic search was performed in PubMed and Embase in December 2019. Studies that met the following criteria were included: (1) the model was applicable in type 2 diabetes; (2) the outcome was retinopathy; and (3) follow-up was more than 1 year. Screening, data extraction (using the checklist for critical appraisal and data extraction for systemic reviews of prediction modelling studies [CHARMS]) and risk of bias assessment (by prediction model risk of bias assessment tool [PROBAST]) were performed independently by two reviewers. Selected models were externally validated in the large Hoorn Diabetes Care System (DCS) cohort in the Netherlands. Retinopathy risk was calculated using baseline data and compared with retinopathy incidence over 5 years. Calibration after intercept adjustment and discrimination (Harrell’s C statistic) were assessed.

**Results:**

Twelve studies were included in the systematic review, reporting on 16 models. Outcomes ranged from referable retinopathy to blindness. Discrimination was reported in seven studies with C statistics ranging from 0.55 (95% CI 0.54, 0.56) to 0.84 (95% CI 0.78, 0.88). Five studies reported on calibration. Eight models could be compared head-to-head in the DCS cohort (*N* = 10,715). Most of the models underestimated retinopathy risk. Validating the models against different severities of retinopathy, C statistics ranged from 0.51 (95% CI 0.49, 0.53) to 0.89 (95% CI 0.88, 0.91).

**Conclusions/interpretation:**

Several prognostic models can accurately predict retinopathy risk in a population-based type 2 diabetes cohort. Most of the models include easy-to-measure predictors enhancing their applicability. Tailoring retinopathy screening frequency based on accurate risk predictions may increase the efficiency and cost-effectiveness of diabetic retinopathy care.

**Registration:**

PROSPERO registration ID CRD42018089122

**Electronic supplementary material:**

The online version of this article (10.1007/s00125-020-05134-3) contains supplementary material, which is available to authorized users.

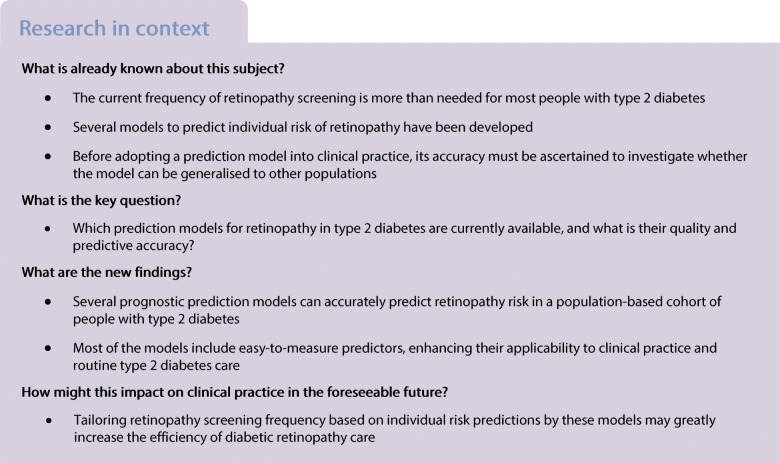



## Introduction

Diabetic retinopathy is a common complication of type 2 diabetes, affecting approximately one-quarter of the type 2 diabetes population [1, 2]. Progression to sight-threatening retinopathy may cause vision loss or blindness, delivering a significant impact on quality of life [3] and high economic burden [4], although this severe end stage occurs in a small fraction of the type 2 diabetes population [2, 5]. Detecting diabetic retinopathy in its early stages through screening efforts allows for medical treatment to prevent or delay loss of vision [6]. Many countries have adopted annual or two-yearly retinopathy screening programmes for people with type 2 diabetes to ensure early diagnosis; these have been effective in reducing progression to severe stages of diabetic retinopathy [7, 8]. However, this frequency of retinopathy screening is often more than needed; most people with type 2 diabetes show no signs of retinopathy at their screening visit and do not require treatment at that time. Because of the growing prevalence of type 2 diabetes, and increased longevity of those with the disease, current annual or two-yearly retinopathy screening frequency is unsustainable, and it becomes increasingly important to manage this complication of type 2 diabetes more efficiently.

Known risk factors for the incidence or progression of diabetic retinopathy include poorly controlled HbA_1c_, high BP and already displaying an early stage of retinopathy [9, 10]. Based on clinical characteristics that are associated with the incidence or progression of retinopathy, several prediction models for retinopathy have been developed [11–13]. These models can be used to estimate the individual retinopathy risk and tailor the screening frequency accordingly, without compromising patient safety [11, 12, 14–16]. Adoption of tailored programmes based on these models could mitigate the burden and costs of over-screening while avoiding the risks from under-screening, thereby providing more effective and cost-effective programmes. Before adopting a prediction model into clinical practice, its accuracy must be ascertained through validation of the model in a population other than the one in which it was developed. This essential step, although often omitted, demonstrates whether the model can be generalised to other populations.

The aim of this study was to systematically review the literature to identify all studies on the development of prognostic prediction models for the risk of developing retinopathy applicable to persons with type 2 diabetes and to determine their quality and predictive accuracy. Subsequently, the selected prediction models were compared head-to-head by validating them in the same large independent population-based cohort of people with type 2 diabetes.

## Methods

We performed a systematic review and an external validation study. The protocol of the systematic review was registered with the International Prospective Register of Systematic Reviews (PROSPERO) on 21 February 2018 (registration no. CRD42018089122), and the review was performed according to the PRISMA-P guideline [17]. The external validation study was reported in line with the transparent reporting of a multivariable prediction model for individual prognosis or diagnosis (TRIPOD) guideline (see electronic supplementary material [ESM] Table 1) [18, 19].

### Systematic review

#### Data sources and searches

The literature was systematically searched for all studies reporting on the development of a prediction model for the risk of developing retinopathy applicable to people with type 2 diabetes. PubMed and Embase were searched from inception to 10 December 2019 using the search string presented in ESM Table 2. Any additional relevant studies were identified by screening the references of the included studies by hand, and related articles were included in the screening process.

#### Study selection for the review

Following the guidance provided in the checklist for critical appraisal and data extraction for systematic reviews of prediction modelling studies (CHARMS) [20]) for defining the review question and study eligibility criteria, studies were eligible for inclusion when meeting the following criteria: (1) the prediction model was developed in people with type 2 diabetes or the general population including diabetes as a predictor; (2) the outcome of the prognostic model was any stage of retinopathy; and (3) the time horizon of the outcome prediction was at least 1 year. A study was excluded when meeting the following criteria: (1) it was performed in animals; (2) it was written in languages other than English or Dutch; (3) it did not address the development of a prediction model; or (4) the prediction model consisted of only one predictor.

#### Data extraction and quality assessment

For the included studies, data extraction was performed using CHARMS [20]. Risk of bias was assessed to identify shortcomings in study design and applicability using the prediction model risk of bias assessment tool (PROBAST) [21, 22]. A combination of two reviewers (of a group of three; A. A. van der Heijden, F. Badloe and J. W. Beulens) independently reviewed each title, abstract and full text and subsequently extracted data and assessed risk of bias and applicability of the included studies.

### External validation

#### Cohort

For external validation of the selected (from the review) prediction models, we used data from the Hoorn Diabetes Care System (DCS) cohort. The DCS cohort is an ongoing population-based cohort including almost all people with type 2 diabetes in the catchment area of the DCS, a primary care setting in the West-Friesland area of the Netherlands [23]. As part of routine diabetes care, participants receive a standardised annual assessment including measurement of diabetes-related risk factors and complications. The resulting database included 13,955 people with type 2 diabetes in 2017, with follow-up ranging from 1 year to 19 years. The study has been approved by the Medical Ethical Review Committee of the VU University Medical Center, Amsterdam. Individuals were informed about the use of their data and were offered the opportunity to opt out. Data were used anonymously. For each individual, year of entry into the cohort was considered baseline. After exclusion of people with insufficient follow-up data on retinopathy status and exclusion of people with the specific outcome of interest at baseline, 10,715 people remained for the prediction of referable diabetic retinopathy, 10,820 for the prediction of sight-threatening retinopathy and 10,874 for the prediction of photocoagulated or proliferative retinopathy. More details can be found in the flowchart shown in ESM Fig. 1.

#### Predictors

According to centrally standardised protocols, examinations and clinical measurements were performed annually. Weight and height were measured when the participant was barefoot and wearing light clothes and BMI was calculated as weight (kg) / height (m^2^). BP was measured twice (3 min apart) after 5 min of rest in a seated position on the right arm using a random-zero sphygmomanometer (Hawksley-Gelman, Lancing, Sussex, UK); from 2003 onwards, an oscillometric device was used (Colin Press-Mate BP-8800 [Komaki City, Japan] and from 2011 onwards, a Welch Allyn ProBP 3400 [Skaneateles Falls, NY, USA]). Using fasting blood, HbA_1c_ was assessed based on the turbidimetric inhibition immunoassay for haemolysed whole EDTA blood, blood glucose level was assessed in fluorinated plasma with the UV test using hexokinase, and levels of triacylglycerols, total cholesterol and HDL-cholesterol were determined enzymatically (Cobas c501; Roche Diagnostics, Mannheim, Germany). LDL-cholesterol concentration was calculated. From an overnight first-voided urine sample, albumin was determined by the reaction of the antigen with anti-albumin antibodies and measured turbidimetrically, and the creatinine concentration in heparinised plasma and urine was determined enzymatically (Cobas c501; Roche Diagnostics). The presence of CVD was based on self-report. Information on medication use was registered yearly by checking the dispensing labels of the medication brought to the monitoring visit. Age at diagnosis was based on registry data. Information on smoking status and ethnic background was obtained by self-report.

#### Retinopathy (outcome)

To determine the presence and stage of retinopathy, participants underwent a 45° fundus photograph of two retinal fields according to the EURODIAB protocol. Retinopathy stage was based on the grading of the worst eye as determined by an experienced ophthalmologist. The grading of retinopathy was performed according to the EURODIAB 0–5 scale as follows: grade 0, no retinopathy; grade 1, minimal non-proliferative retinopathy; grade 2, moderate non-proliferative retinopathy; grade 3, severe non-proliferative or pre-proliferative retinopathy; grade 4, photocoagulated retinopathy; and grade 5, proliferative retinopathy [24]. The ophthalmologist who graded the retinal images did not have access to any other clinical data from the participants. Outcomes used for validation were referable diabetic retinopathy (EURODIAB grade ≥2), sight-threatening diabetic retinopathy (EURODIAB grade ≥3) and photocoagulated or proliferative diabetic retinopathy (EURODIAB grade ≥4).

#### Prediction models selected for validation

Prediction models that were reported in the studies included in the systematic review were all considered for validation. The predictors and outcomes of the models were matched to the variables in the DCS cohort. When the included predictors were not available in the DCS, the predictor was replaced by a suitable proxy variable. When a proxy could not replace the predictor, the model was excluded from validation.

#### Statistical analysis

In the validation cohort, missing data on predictors were imputed using multiple imputation by predictive mean matching, generating five imputed datasets that were pooled using Rubin’s rules. Missing values varied from 0.4% for BMI to 4.0% for albumin/creatinine ratio; 1028 participants (9.6%) had missing data for at least one of the predictors. Baseline characteristics of the study population were presented as proportions or means with SD; variables with a skewed distribution were presented as medians with an interquartile range. For each model that could be applied to the DCS cohort, predicted retinopathy risk was calculated and compared with the incidence of each of the three retinopathy severity outcomes using a 5 year horizon. Performance of the models was estimated using calibration (agreement between predicted and observed retinopathy risk) and discrimination (the ability of the model to distinguish between those who will develop retinopathy and those who will not). Calibration was assessed by visual inspection of calibration plots, the ratio of the observed and expected retinopathy events (O/E), and the Hosmer–Lemeshow *χ*^2^ test. Calibration plots were created by plotting the observed mean retinopathy risk against the predicted mean retinopathy risk within quintiles of the predicted risk. For a well-calibrated risk, the predicted risk would be similar to the observed risk, resulting in an O/E close to 1 and the calibration slope approaching the 45° line of the plot. As most of the intercepts of the models were not reported, and recalibration of models based on the incidence of the outcome of interest in the validation population is recommended, we presented only recalibrated models based on retinopathy incidence in the DCS cohort. For each model, the mean 5 year retinopathy-free survival rate of the DCS cohort was used. Discrimination was assessed for each model using Harrell’s C statistic, accounting for the censored nature of the data. Sensitivity analyses were performed by testing model performance in people without signs of diabetic retinopathy at baseline. Furthermore, the performance of the models was tested applying a 10 year prediction horizon. All analyses were performed with the statistical software package R (version 3.6.1) (www.r-project.org) in combination with the R packages *MICE* (version 3.8.0) and *RMS* (version5.1-4).

## Results

### Systematic review

From the 6907 records, 103 were selected for review of the full text. Of these, 12 met our inclusion criteria, describing the development of 16 prediction models for retinopathy. The flowchart on study inclusion is presented in ESM Fig. 1.

### Characteristics of the models, risk of bias, and applicability

The size of the samples used for model development of the studies included in the systematic review ranged from 295 to 454,575 (ESM Table 3). Two models used blindness as the predicted outcome while the other studies included a less severe form of retinopathy as outcome. The most commonly used prediction time horizons were 5 years and 10 years. The number of events ranged from 61 to 8063. Most of the studies used predictors routinely assessed in people with type 2 diabetes, with HbA_1c_ as the most commonly used predictor (ESM Table 4, ESM Fig. 2).

The majority of the studies scored a high or unclear risk of bias on the analysis domain, with most models not informing or performing on the correct handling of missing data or adjustment for overfitting in model performance (ESM Table 5). The other domains were rated as low risk of bias in 75–100% of the models (ESM Fig. 3). In three studies, high concerns for applicability to our objective were scored due to selective sampling of the participants in the original studies. High applicability was scored for five of the ten studies including participants, predictors and outcomes relevant for a general type 2 diabetes population in a routine care setting.

### Apparent model performance

**Fig. 1:**
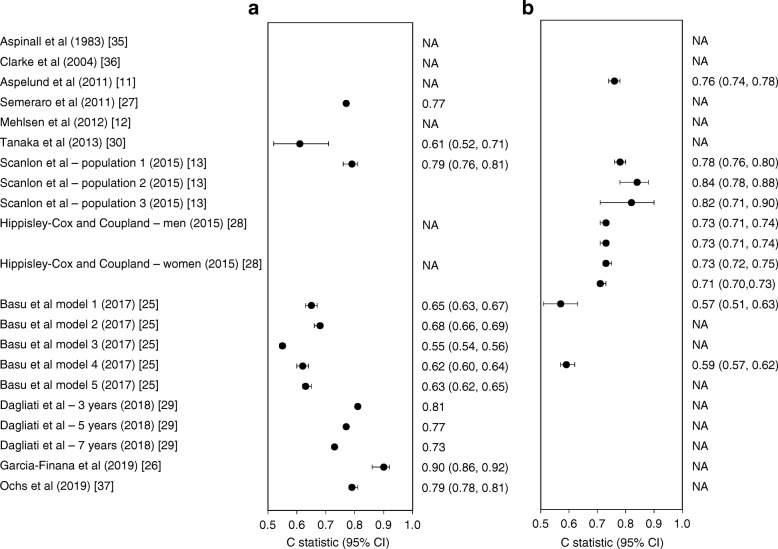
Performance of the models expressed as C statistics resulting from the derivation cohorts (internal validation) (**a**) and external populations (external validation) (**b**) as reported in the model development studies

Information on calibration in the form of a plot or test was reported by four studies based on internal validation and by five studies based on external validation, all showing close agreement between predicted and observed outcomes. Six studies reported C statistics in the derivation cohorts, ranging from 0.55 (95% CI 0.54, 0.56) in Basu et al [25] to 0.90 (95% CI 0.86, 0.92) in Garcia-Finana et al [26] (Fig. 1). In four studies, discriminatory ability in an external population was reported and ranged from 0.57 (95% CI 0.51, 0.63) in Basu et al [25] to 0.84 (95% CI 0.78, 0.88) in Scanlon et al [13]. Four studies lacked information on discrimination and calibration.

### External validation

#### Selection of models for external validation

Of the 12 studies reporting on 16 models, included in the review, eight models were excluded for external validation as the algorithm could not be extracted from the original model or the model mainly included predictors that were not available in the validation cohort and could not be approximated (ESM Table 6). For some models, predictors were approximated to enable validation. For the predictor albuminuria included in the model by Semeraro et al [27], the mean value reported for the derivation cohort was used (12 g/day). Townsend deprivation score, included in the model of Hippisley-Cox and Coupland [28], was not available in the DCS cohort and the reported sex-specific mean value of the deprivation score was used (men 0.5; women 0.8). To predict retinopathy, the model by Scanlon et al [13] used a similar linear predictor but different intercepts in people in whom none, one or two eyes were affected. We estimated the baseline hazard for developing diabetic retinopathy separately for people with and without baseline retinopathy.

#### Characteristics of the external validation cohort

Of the 10,874 people with type 2 diabetes at baseline who were at risk of photocoagulated or proliferative retinopathy, 83 (0.8%) reached this stage of retinopathy during the 5 year prediction horizon. Of the 10,820 people free of sight-threatening retinopathy at baseline, 144 (1.3%) developed sight-threatening retinopathy during follow-up and of the 10,715 people at risk of referable diabetic retinopathy at baseline, 237 (2.2%) developed this condition. In a population free from referable diabetic retinopathy, 493 participants (4.6%) presented with mild retinopathy at baseline. Details of the population are shown in Table 1.

**Table 1:** Baseline characteristics of the Hoorn DCS cohort at risk of any form of referable diabetic retinopathy according to retinopathy status at baseline

Characteristic	No retinopathy	Mild retinopathy^a^
*N*	10,222	493
Age, years	61.7 ± 11.7	63.1 ± 11.9
Men	5516 (54.0)	267 (54.2)
Age at diabetes diagnosis, years	59.2 ± 12.0	57.9 ± 12.6
Time since detection of diabetes, years	0.7 (0.2–7.5)	2.2 (0.4–7.5)
BMI, kg/m^2^	30.4 ± 5.4	29.4 ± 5.2
Systolic BP, mmHg	142 ± 20	146 ± 22
Diastolic BP, mmHg	81 ± 10	83 ± 11
Total cholesterol, mmol/l	5.0 ± 1.2	5.2 ± 1.1
LDL-cholesterol, mmol/l	2.9 ± 1.1	3.1 ± 1.0
HDL-cholesterol, mmol/l	1.2 ± 0.3	1.2 ± 0.3
Triacylglycerol, mmol/l	1.9 ± 1.4	1.9 ± 1.0
Fasting glucose, mol/l	8.3 ± 2.2	9.1 ± 2.7
HbA_1c_, mmol/mol	49.7 (44.3–59.6)	55.2 (46.5–69.4)
HbA_1c_, %	6.7 (6.2–7.6)	7.2 (6.4–8.5)
Currently smoking	2165 (21.2)	111 (22.5)
European descent	9460 (92.6)	439 (89.1)
Antihypertensive medication use	5831 (57.0)	271 (55.0)
Lipid-lowering medication use	4388 (42.9)	163 (33.1)
Glucose-lowering medication use		
None	3211 (31.4)	84 (17.0)
Oral	6244 (61.1)	320 (64.9)
Oral and insulin	417 (4.1)	48 (9.7)
Insulin only	350 (3.4)	41 (8.3)
Kidney failure^b^		
Moderate	1033 (10.1)	68
Severe	29 (0.3)	1 (0.2)
History of CVD^c^	973 (9.5)	41 (8.3)

Data are presented as means±SD, median (interquartile range) or *n* (%)

^a^Mild retinopathy, EURODIAB grade 1

^b^Moderate kidney failure, eGFR 30–60 ml min^−1^ [1.73 m]^2^; severe kidney failure, eGFR ≤30 ml min^−1^ [1.73 m]^2^

^c^History of CVD includes myocardial infarction, heart failure and stroke

#### Calibration

Considering the performance of the models specifically for predicting the retinopathy outcome (as they were developed for), the calibration plots display the relationship between the predicted risk and observed retinopathy incidence of the eight prediction models after recalibration based on retinopathy incidence in the DCS cohort (ESM Fig. 4, ESM Table 7). In most of the models, the first quintiles showed agreement between the predicted risk and observed retinopathy incidence, and an underestimation of retinopathy risk in people in the highest risk quintile. The calibration plots of Semeraro et al [27] and Scanlon et al [13] showed better calibration. The model of Dagliati et al [29] showed less agreement between the predicted and observed risks within lower quintiles. The O/E ratios that showed the most substantial deviation from 1 were from the models of Aspelund et al [11] and Dagliati et al [29], mainly underestimating retinopathy risk. All models showed *p* values <0.05 for the Hosmer–Lemeshow *χ*^2^ test.

#### Discrimination

**Fig. 2:**
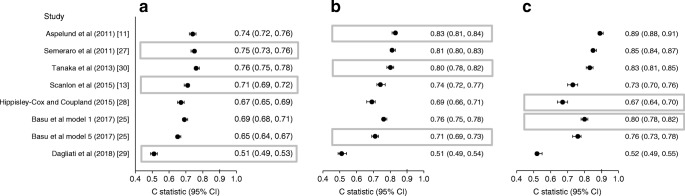
Discriminative ability of the models for prediction of referable diabetic retinopathy (EURODIAB grade ≥2) (**a**), sight-threatening diabetic retinopathy (EURODIAB grade ≥3) (**b**) and photocoagulated or proliferative diabetic retinopathy (EURODIAB grade ≥4) (**c**) in the DCS cohort. Results are presented as C statistics (95% CI). The grey boxes indicate the models intended to predict the retinopathy stage shown in that figure part

In Fig. 2, the discriminatory ability of the models selected for validation is presented for each of the three retinopathy outcome measures. Considering the specific retinopathy stage used in model development, C statistics ranged from 0.51 (95% CI: 0.49, −0.53) to 0.83 (95% CI: 0.81, −0.84). Applying each model to all three retinopathy outcomes improved discriminatory power for higher severities of retinopathy in most models, and C statistics ranged from 0.51 (95% CI 0.49, 0.53) to 0.89 (95% CI 0.88, 0.91). For all three retinopathy outcomes, the models by Tanaka et al [30] Semeraro et al [27] and Aspelund et al [11] showed the highest C indices (Fig. 2).

#### Sensitivity analyses

When the prediction horizon used for the external validation was extended to 10 years, a slight decrease in performance was seen in most of the models (ESM Table 8). Applying the model in people without any signs of retinopathy at baseline resulted in the largest decrease in C statistics in the models that include baseline presence of diabetic retinopathy as a predictor (ESM Table 9).

## Discussion

The current study provides a comprehensive overview of the available prognostic prediction models for developing retinopathy (beyond 1 year) in people with type 2 diabetes. We identified 12 studies describing the development of 16 prediction models with outcomes ranging from any form of retinopathy to blindness. Eight of the 16 models could be validated and directly compared head-to-head in a large independent validation cohort. Notably, we found that four models (i.e. Semeraro et al [27], Scanlon et al [13], Aspelund et al [11] and Tanaka et al [30]) showed a better calibration plus discrimination than the other four.

This systematic review demonstrated that most of the models used predictors that were obtained in routine diabetes care settings, increasing their applicability to daily practice. According to the recently published PROBAST tool, most of the models showed an overall high or unclear risk of bias, mainly caused by vague reporting on model development (domain 4). This might be due to the fact that the TRIPOD guideline for transparent reporting of prediction model studies is relatively new and was not available during the development of most of the models included in the review.

Considering the calibration of the models, even after recalibration to retinopathy incidence in the DCS cohort, most models still showed overestimation for individuals in the highest risk quintile for retinopathy. Calibration seemed best in the model of Semeraro et al [27] and Scanlon et al [13] When comparing the results of several previous external validation studies of the model of Aspelund et al [11, 14, 15], similar or slightly better discrimination was observed in the DCS cohort. External validation in the DCS cohort yielded similar results for discriminative performance after internal validation of the model of Semeraro et al [27]. For the model of Tanaka et al, better discrimination was found in the external validation compared with the models’ internal validation [30]. In this validation study, we found lower discriminatory power for the models of Scanlon et al and Hippisley-Cox and Coupland, which presented slightly lower C statistics compared with the external validation of these two models in different cohorts [13, 28]. Reasons for this lower discrimination may be the absence of some of the models’ predictor variables in the DCS cohort. The model of Scanlon et al [13] distinguishes for the presence of mild retinopathy in one or two eyes in the baseline hazard. Whether one or both eyes were affected at baseline was not recorded in the DCS data. Additionally, the discriminatory ability of Scanlon’s model improved after the exclusion of people with signs of retinopathy at baseline. The model of Hippisley-Cox and Coupland [28] used blindness as the predicted outcome; this information was not available in the DSC cohort. Photocoagulated or proliferative retinopathy was used as a proxy for this outcome. Townsend deprivation score was also not available in the DCS cohort, leaving cholesterol/HDL-cholesterol ratio, diabetes duration and presence of chronic renal disease as the predictor variables, which may have affected model performance. The model of Basu et al showed better discriminatory power after external validation in the DCS cohort, compared with previous external validation studies [25, 31]. The model of Basu et al had the highest number of predictors. Besides common predictors such as HbA_1c_, lipids and BP, the model includes predictors on glucose-lowering medication use, history of CVD, and renal function [25]. The model was developed using data from people with type 2 diabetes selected based on their unfavourable risk profile. Application of the model in our relatively well-controlled population showed that the model was capable of discriminating between low and high risk. For the model of Dagliati et al [29], a substantial decrease in discriminative ability was seen between the model’s internal validation and the DCS cohort. The small sample size in the original population and the low number of events might have resulted in an overfitted model that performed less well in our large cohort.

A limitation of the systematic review is that some prediction models may have been missed due to language restrictions. A limitation of the external validation study is the low incidence of retinopathy in the DCS cohort. Recent publications indicate that studies aiming to test model performance require at least 100 events [32, 33]. For referable and sight-threatening retinopathy, this criterion is fulfilled. For the more severe stages, the analysis might have been underpowered. However, we observed a stable trend in the discriminatory power with increasing severity of retinopathy, possibly indicating that the results would not be affected largely by the lower number of end-stage retinopathy events. Another limitation is that the study population was primarily of European descent, hindering the extrapolation to populations with a different migration background. The model of Scanlon et al was previously validated in cohorts with people with African Caribbean and South Asian backgrounds and showed similar accuracy as in white populations [13]. An additional limitation is that not all predictors used by the models were available in the DCS cohort.

Several study strengths should be noted. First, the results of the validation study apply to routine clinical practice settings for people with type 2 diabetes. Second, the use of a large, unselected cohort of people with type 2 diabetes, including almost all people with type 2 diabetes in the catchment area of the DCS, enhances this external validation study. Third, all predictor and outcome measurements in the cohort were performed according to centrally standardised protocols, including consistency in measuring and defining the presence and grade of retinopathy; this enhanced the reliability of the data and resulted in a low number of missing variables.

In recent literature, approaches have been suggested for reducing the frequency of unneeded retinopathy screening; these include extending the screening interval from 1 year to 2 years for people who are at low risk of retinopathy, defined by two subsequent retinopathy examinations without signs of retinopathy [34]. However, prediction models for retinopathy more efficiently use available clinical data to identify people at low risk, by providing an individual risk estimation. This study showed that several models are capable of distinguishing between those at low risk and those at high risk for retinopathy and can be used to tailor the screening frequency. Adequate performance of a model based on calibration as well as discrimination is a prerequisite for its clinical relevance. When using a model to tailor screening intervals, overestimating or underestimating the absolute risk may lead to a too frequent or infrequent screening respectively, which may be overcome by model recalibration. Once this is evaluated, clinical applicability is leading for the choice of a suitable model. Based on the results on discrimination and calibration after external validation of the models in the DCS cohort, four models performed well enough and can help facilitate tailored retinopathy care for people with type 2 diabetes. Two of these models do not include current retinopathy status as a predictor, which might seem counterintuitive from a clinical perspective. The models by Scanlon et al [13] and Aspelund et al [11] include information on current retinopathy in addition to routinely collected risk factors and predict earlier stages of retinopathy, enhancing their applicability. In the DCS we already have experience tailoring retinopathy care after implementation of the model developed by Aspelund et al. This model additionally provides a tailored screening interval ranging from 6 months to a maximum of 5 years based on the estimated retinopathy risk and hence is instrumental in adapting imaging frequency to personal risk, enhancing efficient use of medical resources. Previous studies showed that this method for personalising screening frequency is safe and reduces the average screening frequency [14–16].

To conclude, this comprehensive systematic review and head-to-head comparison of all existing prognostic models predicting the risk of developing retinopathy in individuals with type 2 diabetes, shows that several existing prediction models can accurately predict retinopathy risk in a population-based cohort of people with type 2 diabetes. The models of Scanlon et al [13] and Aspelund et al [11] performed best overall and contain easy-to-measure predictors, increasing their suitability for clinical practice. Tailoring retinopathy screening frequency based on individual risk predictions by these models may highly increase the efficiency of diabetic retinopathy care.

## Electronic supplementary material

ESM (PDF 705 kb)

## Data Availability

The steering committee of the Hoorn studies will consider reasonable requests for the sharing of de-identified patient-level data. Requests should be made to the corresponding author.

## Abbreviations

CHARMS: Checklist for critical appraisal and data extraction for systemic reviews of prediction modelling studies
DCS: Diabetes Care System
O/E: Ratio of the observed and expected retinopathy events
PROBAST: Prediction model risk of bias assessment tool
TRIPOD: Transparent reporting of a multivariable prediction model for individual prognosis or diagnosis
