# Supplementary material for: Prediction models for development of retinopathy in people with type 2 diabetes: systematic review and external validation in a Dutch primary care setting
Source: Diabetologia. 2020 Apr 3;63(6):1110–9. doi: 10.1007/s00125-020-05134-3 (PMC7228897; doi:10.1007/s00125-020-05134-3)
Supplement: Supplementary file 1 — (PDF 705 kb) [file 125_2020_5134_MOESM1_ESM.pdf]

SUPPLEMENTARY DATA

ESM Table 1. TRIPOD Checklist for Prediction Model Validation

Section/Topic	Item	Checklist Item	Page
**Title and abstract**			
Title	1	Identify the study as developing and/or validating a multivariable prediction model, the target population, and the outcome to be predicted.	1
Abstract	2	Provide a summary of objectives, study design, setting, participants, sample size, predictors, outcome, statistical analysis, results, and conclusions.	2
**Introduction**			
Background and objectives	3a	Explain the medical context (including whether diagnostic or prognostic) and rationale for developing or validating the multivariable prediction model, including references to existing models.	3
	3b	Specify the objectives, including whether the study describes the development or validation of the model or both.	3
**Methods**			
Source of data	4a	Describe the study design or source of data (e.g., randomized trial, cohort, or registry data), separately for the development and validation data sets, if applicable.	5, Table 1
	4b	Specify the key study dates, including start of accrual; end of accrual; and, if applicable, end of follow-up.	5
Participants	5a	Specify key elements of the study setting (e.g., primary care, secondary care, general population) including number and location of centres.	5
	5b	Describe eligibility criteria for participants.	5
	5c	Give details of treatments received, if relevant.	NA
Outcome	6a	Clearly define the outcome that is predicted by the prediction model, including how and when assessed.	5
	6b	Report any actions to blind assessment of the outcome to be predicted.	5
Predictors	7a	Clearly define all predictors used in developing or validating the multivariable prediction model, including how and when they were measured.	5,6
	7b	Report any actions to blind assessment of predictors for the outcome and other predictors.	5
Sample size	8	Explain how the study size was arrived at.	5, Figure 1
Missing data	9	Describe how missing data were handled (e.g., complete-case analysis, single imputation, multiple imputation) with details of any imputation method.	6
Statistical analysis methods	10c	For validation, describe how the predictions were calculated.	6,7
	10d	Specify all measures used to assess model performance and, if relevant, to compare multiple models.	6,7
	10e	Describe any model updating (e.g., recalibration) arising from the validation, if done.	7
Risk groups	11	Provide details on how risk groups were created, if done.	7
Development vs. validation	12	For validation, identify any differences from the development data in setting, eligibility criteria, outcome, and predictors.	8, Supplemental table
**Results**			

Participants	13a	Describe the flow of participants through the study, including the number of participants with and without the outcome and, if applicable, a summary of the follow-up time. A diagram may be helpful.	8, Figure 1
	13b	Describe the characteristics of the participants (basic demographics, clinical features, available predictors), including the number of participants with missing data for predictors and outcome.	6, Figure 1, Supplemental Table 7
	13c	For validation, show a comparison with the development data of the distribution of important variables (demographics, predictors and outcome).	-
Model performance	16	Report performance measures (with CIs) for the prediction model.	9, Figure 3, Figure 4
Model-updating	17	If done, report the results from any model updating (i.e., model specification, model performance).	Figure 4
**Discussion**			
Limitations	18	Discuss any limitations of the study (such as non-representative sample, few events per predictor, missing data).	10,11
Interpretation	19a	For validation, discuss the results with reference to performance in the development data, and any other validation data.	11
	19b	Give an overall interpretation of the results, considering objectives, limitations, results from similar studies, and other relevant evidence.	11,12
Implications	20	Discuss the potential clinical use of the model and implications for future research.	12
**Other information**			
Supplementary information	21	Provide information about the availability of supplementary resources, such as study protocol, Web calculator, and data sets.	√
Funding	22	Give the source of funding and the role of the funders for the present study.	11

From: Collins GS, Ogundimu EO, Altman DG. Sample size considerations for the external validation of a multivariable prognostic model: a resampling study. Stat Med 2016;35(2):214-26. doi: 10.1002/sim.6787

ESM Table 2. Search string

PUBMED

(("Retinal Diseases"[Mesh] OR "Vision Disorders"[Mesh] OR blindness[tiab] OR retinopath*[tiab] OR vision impairement[tiab] OR visual impairement[tiab] OR vision disorder*[tiab] OR visual disorder*[tiab]) AND (Validat*[tiab] OR validit*[tiab] OR Predict*[tiab] OR Rule*[tiab] OR (Decision*[tiab] AND (Model*[tiab] OR Clinical[tiab])) OR (Prognostic[tiab] AND (History[tiab] OR Variable*[tiab] OR Criteria[tiab] OR Score[tiab] OR Scores*[tiab] OR Characteristic*[tiab] OR Finding*[tiab] OR Factor*[tiab] OR Model*[tiab])) OR risk score*[tiab] OR risk assessment*[tiab] OR algorithm*[tiab]) AND ("Diabetes Mellitus"[Mesh] OR diabetes[tiab] OR (diabetic*[tiab] AND (non insulin depend*[tiab] OR noninsulin depend*[tiab] OR noninsulindepend*[tiab] OR non insulindepend*[tiab])) OR dm2[tiab] OR niddm[tiab] OR dm 2[tiab] OR t2d*[tiab] OR dm type 2[tiab] OR type 2 diabet*[tiab] OR type two diabet*[tiab] OR type II diabet*[tiab] OR dm type II[tiab])) NOT ("Animals"[Mesh] NOT "Humans"[Mesh])

EMBASE

'retina disease'/exp OR 'visual disorder'/exp OR blindness:ab,ti OR retinopath*:ab,ti OR 'vision impairement':ab,ti OR 'visual impairement':ab,ti OR 'vision disorder*':ab,ti OR 'visual disorder*':ab,ti

AND

validat*:ab,ti OR validit*:ab,ti OR predict*:ab,ti OR rule*:ab,ti OR (decision* NEAR/3 (model* OR clinical)):ab,ti OR (prognostic NEAR/3 (history OR variable* OR criteria OR score OR scores* OR characteristic* OR finding* OR factor* OR model*)):ab,ti OR 'risk score*':ab,ti OR 'risk assessment*':ab,ti OR algorithm*:ab,ti

AND

'diabetes mellitus'/exp OR diabetes:ab,ti OR (diabetic* NEAR/3 ('non insulin depend*' OR 'noninsulin depend*' OR noninsulindepend* OR 'non insulindepend*')):ab,ti OR dm2:ab,ti OR niddm:ab,ti OR 'dm 2':ab,ti OR t2d*:ab,ti OR 'dm type 2':ab,ti OR 'type 2 diabet*':ab,ti OR 'type two diabet*':ab,ti OR 'type ii diabet*':ab,ti OR 'dm type ii':ab,ti

NOT ([animals]/lim NOT [humans]/lim)

ESM Table 3. Characteristics of the prediction models for retinopathy in type 2 diabetes

Reference	Origin cohort	Population	Sample size	Events	Model	Prediction horizon	Outcome
Aspinall et al (1983) [1]	Scotland	T1D/T2D	295	86	Logistic	7 years	Any form of retinopathy
Clarke et al (2004) [2]	UK	T2D	3,642	NR	Weibull	lifetime	Blindness
Aspelund et al (2011) [3]	Iceland, UK, USA	T1D/T2D	T1D: 634<br>T2D: 929	T1D: 235<br>T2D: 61	Weibull	5 years	DME or PDR
Semeraro et al (2011)[4]	Italy	T2D	5,034	569	Cox	1,2,3,4 years	DR (not further specified)
Mehlsen et al (2012) [5]	Denmark	T1D/T2D	T1D: 1,275<br>T2D: 3,572	T1D:171<br>T2D:388	Logistic	3 years	Treatment of DME/PDR
Tanaka et al (2013) [6]	Japan	T2D	1,748	415	Cox	5 years	Intraretinal microvascular abnormalities and venous changes/ New vessels vitreous haemorrhage, fibrous proliferation and retinal detachment
Scanlon et al (2015) [7]	UK	T1D/T2D	7,012	606	Cox	5 years	Referable DR and Referable diabetic maculopathy
Hippisley-Cox and Coupland (2015) [8]	UK	T1D/T2D	454,575	8,063	Cox	10 years	Blindness
Basu et al (2017) [9]							
- Model 1	USA/ Canada	T2D	9,635	901	Cox	10 years	Retinopathy requiring photocoagulation or vitrectomy
- Model 2	USA/ Canada	T2D	9,635	1,476	Cox	10 years	Cataract extraction
- Model 3	USA/ Canada	T2D	9,635	3,559	Cox	10 years	Three-line reduction in visual acuity
- Model 4	USA/ Canada	T2D	9,635	776	Cox	10 years	Severe vision loss (<20/200 visual acuity by Snellen chart)
- Model 5	USA/ Canada	T2D	9,635	168	Cox	10 years	Composite of photocoagulation, vitrectomy, or severe vision loss
Dagliati et al (2018) [10]	Italy	T2D	943	118	Logistic	3, 5, and 7 years	Specific lesions at dilated fundoscopy
Garcia-Finana et al (2019)[11]	UK	T1D/T2D	T1D: 651<br>T2D: 12,452	T1D: 66<br>T2D: 275	Generalised linear mixed-effects model and Markov chain Monte Carlo model	1 year	Moderate/severe pre-proliferative DR or proliferative DR and/or maculopathy
Ochs et al (2019) [12]	UK	T1D/T2D	T1D: 19,070<br>T2D: 220,276	NR	Generalised linear models and survival models	5 years	Referable DR: ETDRS scale retinopathy grade 3 or 4, or maculopathy grade 2

T1D: type 1 diabetes; T2D: type 2 diabetes; DME: diabetic macular oedema; PDR: proliferative diabetic retinopathy; DR: diabetic retinopathy; ETDRS: Early Treatment Diabetic Retinopathy Study

ESM Table 4. Predictors in the models included in the systematic review

	Aspinall et al (1983) [1]	Clarke et al (2004() [2]	Aspelund et al (2011) [3]	Semeraro et al (2011_[4]	Mehlsen et al (2012) [5]	Tanaka et al (2013) [6]	Scanlon et al (2015) [7]	Hippisely-Cox and coupland (2015) [8]	Basu et al model 1 (2017) [9]	Basu et al model 5 (2017) [9]	Dagliati et al (2018) [10]	Garcia-Finana et al (2019) [11]	Ochs et al (2019) [12]
**Sociodemographic**													
Age		•				•			•	•			•
Sex			•	•	•			•	•	•			•
Deprivation								•					
Ethnicity									•	•			
**Lifestyle**													
Smoking status											•		
BMI						•					•		
Age at diagnosis					•								
Diabetes duration	•		•	•	•	•	•	•				•	•
Type of diabetes			•					•				•	
Postprandial blood glucose	•												
**Biomedical**													
HbA1c		•	•	•	•	•	•		•	•	•	•	•
Systolic blood pressure			•	•					•	•		•	
Total cholesterol							•		•	•			•
HDL									•	•			
Cholesterol/HDL ratio								•					
Serum creatinine							•		•	•			
Albumin-creatinine ratio						•			•				
Albuminuria				•									
Creatinine clearance				•									
**Comorbidities**													
History of CVD									•	•			
Chronic renal disease								•					
Proteinuria	•												
**Medication use**													
Antihypertensive drugs									•	•	•		
Glucose lowering drugs				•					•	•			
**Eye exam**													
Presence of retinopathy			•				•	•				•	•
Colour discrimination	•												
Hard exudates					•								
Haemorrhages					•								
Other													
Attendance previous appointment												•	

ESM Table 5. Results of the assessment of risk of bias and concerns for applicability

	**Risk of Bias**				
	**participants**	**predictors**	**outcome**	**analysis**	**overall Risk of Bias**
Aspinall et al (1983) [1]	+	+	?	-	high
Clarke et al (2004) [2]	+	+	+	?	unclear
Aspelund et al (2011) [3]	+	+	+	?	unclear
Semeraro et al (2011) [4]	+	+	+	-	high
Mehlsen et al (2012) [5]	+	+	+	?	unclear
Tanaka et al (2013) [6]	-	+	+	?	high
Scanlon et al (2015) [7]	+	+	+	?	unclear
Hippisley-Cox and Coupland (2015) [8]	+	+	+	?	unclear
Basu et al (2017) [9]	-	+	+	+	high
Dagliati et al (2018) [10]	-	+	+	?	high
Garcia-Finana et al (2019) [11]	+	+	+	-	high
Ochs et al (2019) [12]	+	+	+	-	high
	**Applicability**				
	**participants**	**predictors**	**outcome**		**Overall concerns for applicability**
Aspinall et al (1983) [1]	+	-	+		high
Clarke et al (2004) [2]	-	+	+		high
Aspelund et al (2011) [3]	+	+	+		low
Semeraro et al (2011)[4]	+	+	+		low
Mehlsen et al (2012) [5]	+	-	+		high
Tanaka et al (2013) [6]	-	+	+		high
Scanlon et al (2015) [7]	+	+	+		low
Hippisley-Cox and Coupland (2015) [8]	+	+	+		low
Basu et al (2017) [9]	-	+	+		high
Dagliati et al (2018) [10]	+	+	+		low
Garcia-Finana et al (2019)[11]	+	+	+		low
Ochs et al (2019) [12]	+	+	+		low

+ indicates low risk of bias/low concern regarding applicability; − indicates high risk of bias /high concern regarding applicability; and ? indicates unclear risk of bias /unclear concern regarding applicability.

ESM **Table 6.** Models excluded for validation in the Diabetes Care System cohort

**Model**	**Reason for exclusion**
Aspinall et al (1983) [1]	The main predictor variables are not available in the DCS cohort: Yellow-blue color discrimination, Blood glucose control: mean of previous six postprandial blood glucose values, and proteinuria (not further specified).
Clarke et al (2004) [2]	The algorithm for retinopathy is part of larger simulation model, and could not be applied to the Diabetes Care System cohort
Mehlsen et al (2012) [5]	The model mainly uses variables that are not available in the Diabetes Care System cohort: number of retinal haemorrhages, number of hard eduxates
Basu et al, model 2-4 (2017) [9]	Three of the five models of Basu use outcomes that are less applicable to the DCS cohort: cataract extraction, three-line reduction in visual acuity, or severe vision loss
Garcia-Finana et al (2019)[11]	Application to the Diabetes Care System cohort was limited due to model design.
Ochs et al (2019) [12]	Application to the Diabetes Care System cohort was limited due the inclusion of variables containing information on retinopathy in both eyes.

ESM Table 7. Number of observed retinopathy events for each quintile of predicted risk

	Q1	Q2	Q3	Q4	Q5
**EURODIAB grade ≥ 2**					
Semeraro et al (2011)[4]	11	21	33	33	139
Scanlon et al (2015) [7]	6	13	22	23	173
Dagliati et al (2018) [10]	41	62	35	35	64
**EURODIAB grade ≥ 3**					
Aspelund et al (2011) [3]	5	6	9	20	104
Tanaka et al (2013) [6]	5	8	12	22	97
**EURODIAB grade ≥ 4**					
Hippisley-Cox and Coupland (2015) [8]	7	14	13	8	41
Basu et al, model 1 (2017) [9]	2	4	4	16	57
Basu et al, model 5 (2017) [9]	2	7	9	18	47

ESM Table 8. Performance of the models in the Diabetes Care System cohort using a ten-year horizon

	**Referable retinopathy (312 cases)**	**Sight-threatening retinopathy (181 cases)**	**Photocoagulated/ proliferative retinopathy (118 cases)**
Aspelund et al (2011) [3]	0.72 (0.71, 0.74)	0.81 (0.80, 0.98)	0.88 (0.87, 0.89)
Semeraro et al (2011)[4]	0.73 (0.72, 0.75)	0.80 (0.78, 0.81)	0.84 (0.83, 0.86)
Tanaka et al (2013) [6]	0.75 (0.74, 0.76)	0.80 (0.78, 0.82)	0.83 (0.81, 0.85)
Scanlon et al (2015) [7]	0.70 (0.68, 0.71)	0.75 (0.73, 0.77)	0.75 (0.72, 0.77)
Hippisley-Cox and Coupland (2015) [8]	0.66 (0.65, 0.68)	0.69 (0.67, 0.71)	0.69 (0.66, 0.71)
Basu et al, model 1 (2017) [9]	0.68 (0.67, 0.70)	0.75 (0.73, 0.77)	0.79 (0.77, 0.81)
Basu et al, model 5 (2017) [9]	0.64 (0.63, 0.66)	0.69 (0.68, 0.71)	0.74 (0.72, 0.76)
Dagliati et al (2018) [10]	0.50 (0.49, 0.52)	0.50 (0.48, 1.52)	0.51 (0.48, 0.53)

Discriminative ability of the models for prediction of referable diabetic retinopathy (Eurodiab grade ≥ 2), sight-threatening diabetic retinopathy (Eurodiab grade ≥ 3), and photocoagulated of proliferative diabetic retinopathy (Eurodiab grade ≥ 4) in the Diabetes Care System cohort. Results are presented as C-statistics (95% CI).

ESM Table 9. Performance of the models in the Diabetes Care System according to presence of retinopathy signs at baseline

	**Referable retinopathy**		**Sight-threatening retinopathy**		**Photocoagulated/ proliferative retinopathy**	
	**No DR**	**DR**	**No DR**	**DR**	**No DR**	**DR**
N	10,222	493	10,222	598	10,222	652
Cases (%)	141 (1.4%)	96 (19.5%)	58 (0.6%)	86 (14.4%)	17 (0.2%)	66 (10.1%)
Aspelund et al (2011) [3]	0.67 (0.65, 0.69)	0.60 (0.58, 0.63)	0.70 (0.69, 0.74)	0.70 (0.68, 0.73)	0.76 (0.72, 0.80)	0.72 (0.70, 0.74)
Semeraro et al (2011)[4]	0.72 (0.70, 0.74)	0.67 (0.64, 0.69)	0.74 (0.71, 0.77)	0.73 (0.71, 0.76)	0.85 (0.81, 0.89)	0.72 (0.69, 0.74)
Tanaka et al (2013) [6]	0.75 (0.73, 0.77)	0.66 (0.63, 0.68)	0.75 (0.72, 0.78)	0.72 (0.69, 0.74)	0.86 (0.83, 0.90)	0.69 (0.67, 0.72)
Scanlon et al (2015) [7]	0.74 (0.72, 0.76)	0.66 (0.63, 0.69)	0.76 (0.73, 0.79)	0.73 (0.71, 0.76)	0.84 (0.79, 0.89)	0.70 (0.67, 0.73)
Hippisley-Cox and Coupland (2015) [8]	0.65 (0.63, 0.62)	0.60 (0.56, 0.64)	0.63 (0.59, 0.67)	0.61 (0.56, 0.66)	0.65 (0.58, 0.72)	0.57 (0.51, 0.62)
Basu et al, model 1 (2017) [9]	0.68 (0.66, 0.71)	0.61 (0.59, 0.64)	0.72 (0.68, 0.75)	0.70 (0.67, 0.72)	0.80 (0.75, 0.84)	0.70 (0.68, 0.73)
Basu et al, model 5 (2017) [9]	0.69 (0.64, 0.68)	0.57 (0.54, 0.59)	0.68 (0.65, 0.71)	0.64 (0.61, 0.66)	0.74 (0.69, 0.79)	0.66 (0.64, 0.69)
Dagliati et al (2018) [10]	0.52 (0.50, 0.55)	0.47 (0.45, 0.50)	0.51 (0.47, 0.54)	0.50 (0.48, 0.53)	0.54 (0.47, 0.61)	0.51 (0.48, 0.55)

DR=diabetic retinopathy. Discriminative ability of the models for prediction of referable diabetic retinopathy (Eurodiab grade ≥ 2). sight-threatening diabetic retinopathy (Eurodiab grade ≥ 3). and photocoagulated of proliferative diabetic retinopathy (Eurodiab grade ≥ 4) in the Diabetes Care System cohort. Results are presented as C-statistics and 95% confidence intervals.

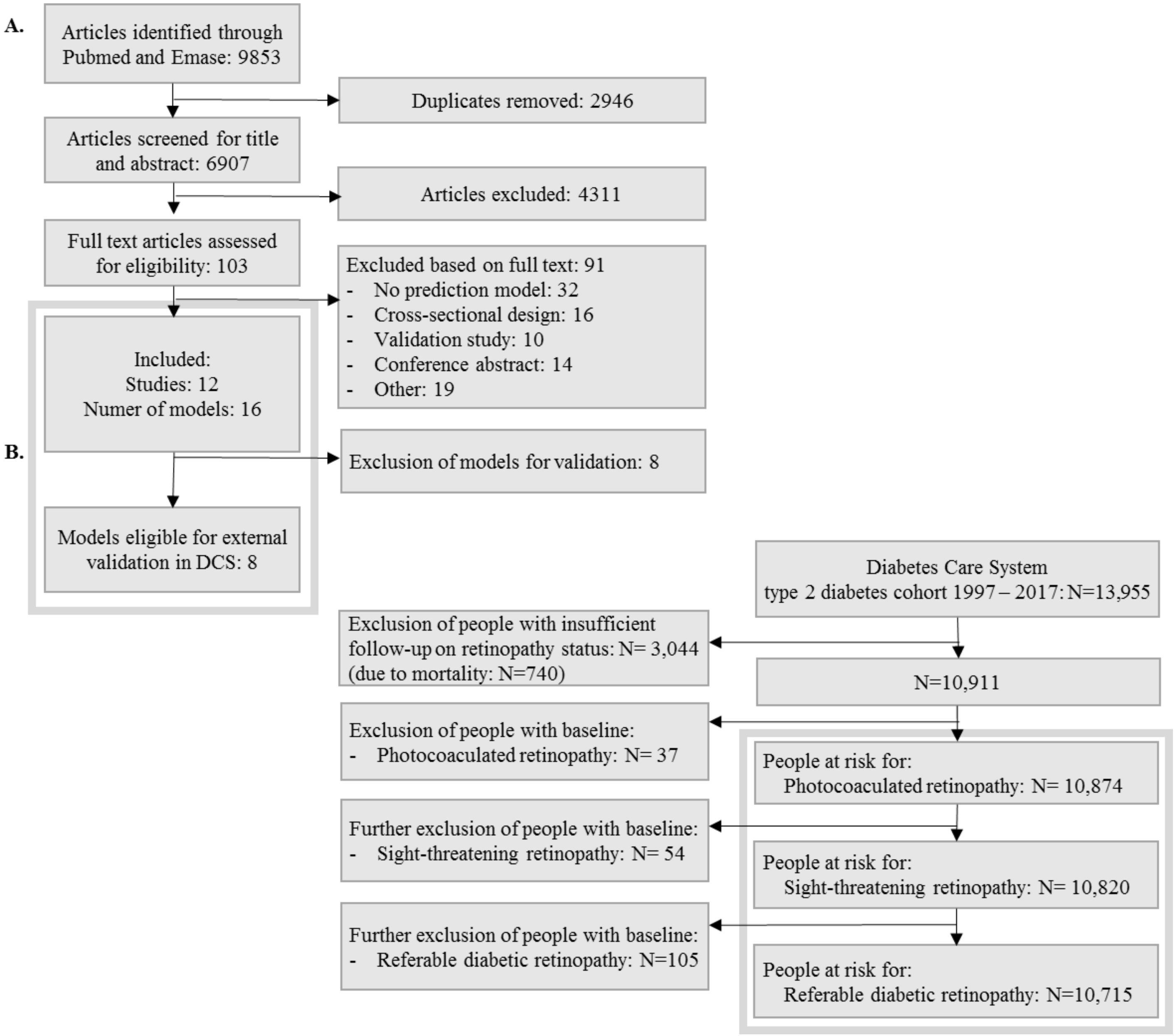

ESM Figure 1. Flow diagram of selection of studies that described the development of a prediction model for the risk of retinopathy based on a systematic literature search (A), and the selection of studies and study population for external validation (B)

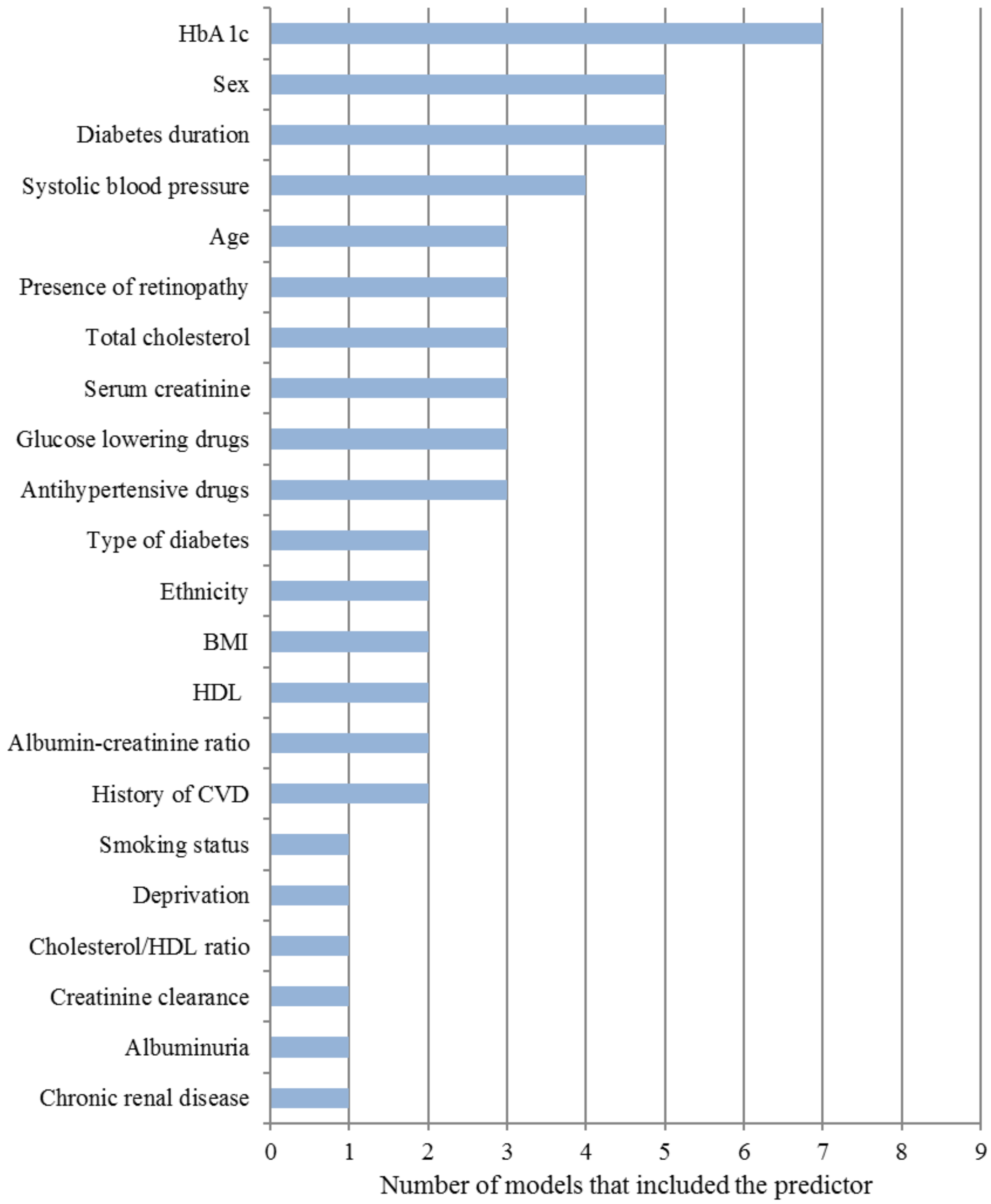

ESM Figure 2. Overview of predictors included in the retinopathy models selected for external validation

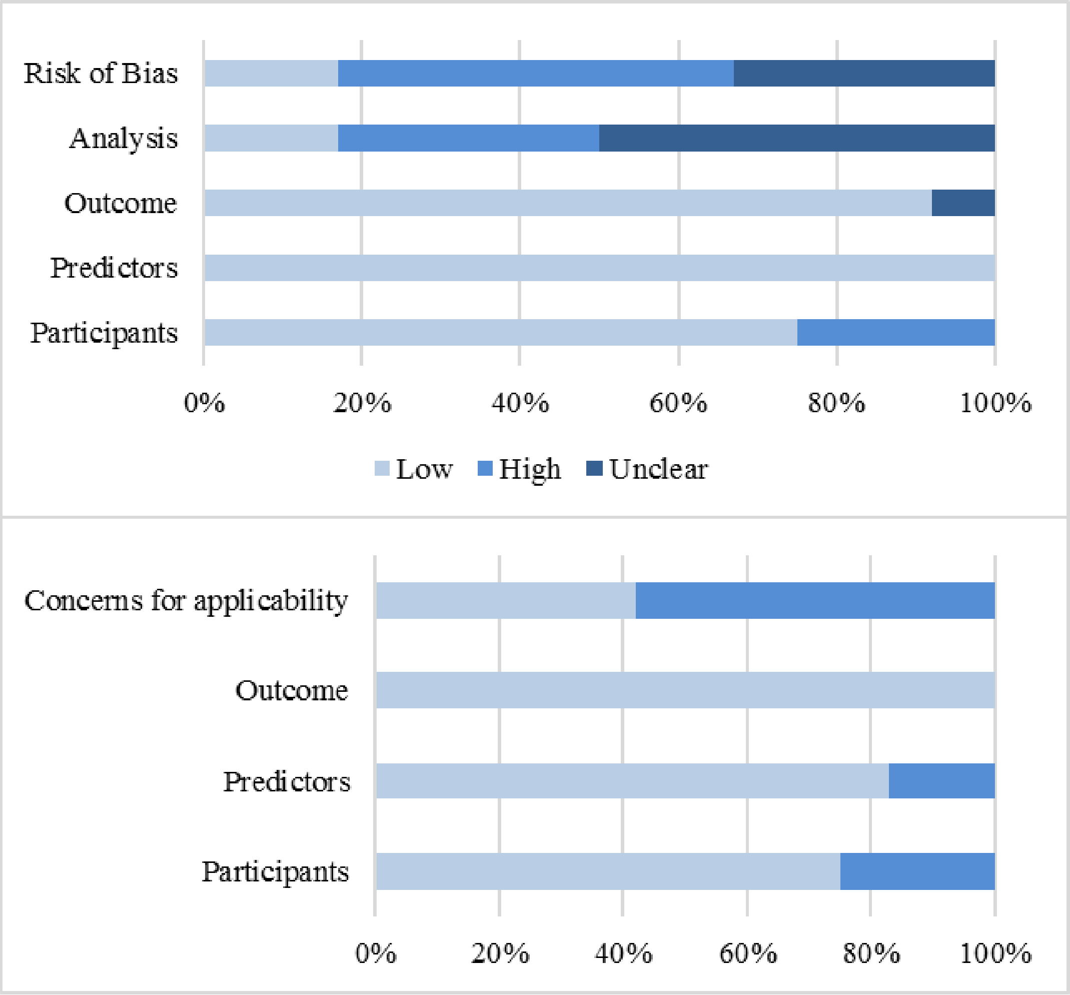

ESM Figure 3. Summary of the risk of bias and concerns for applicability of the retinopathy prediction models

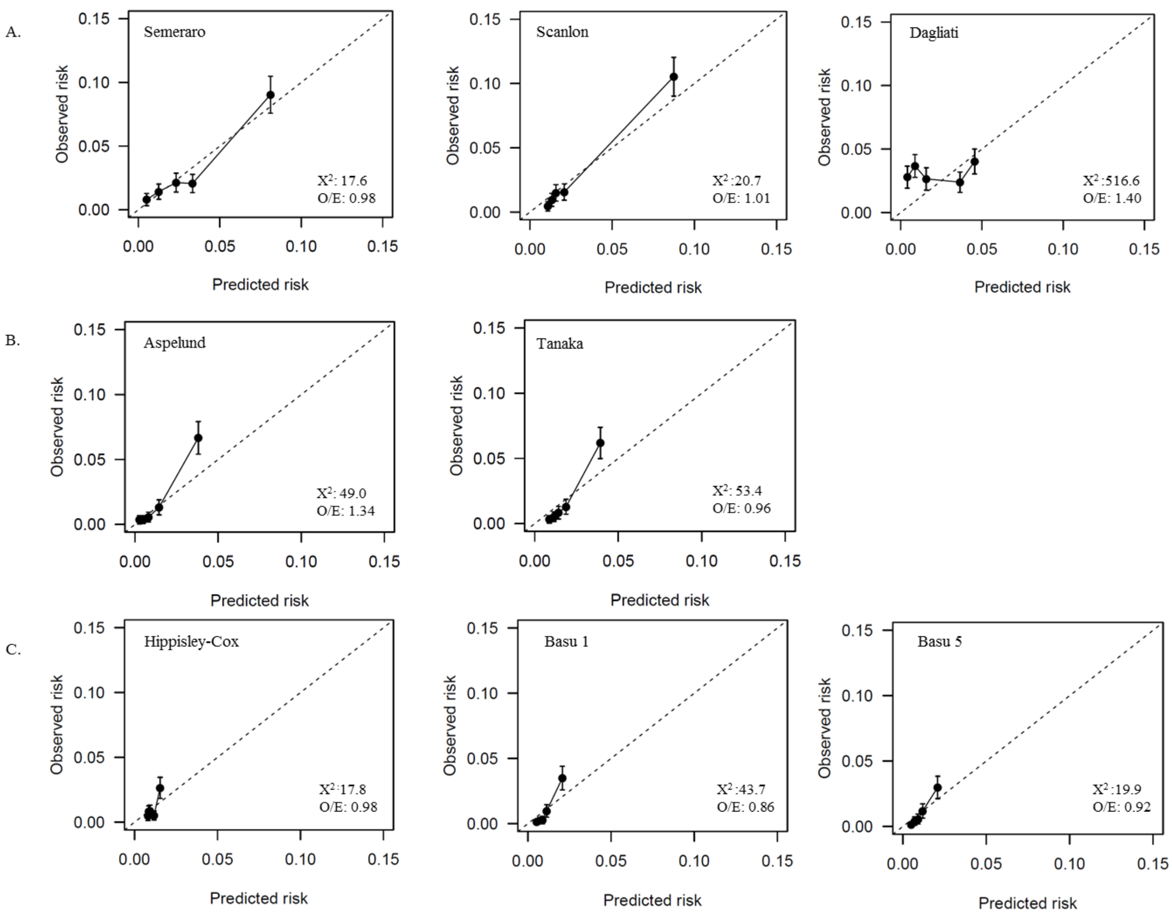

ESM Figure 4. Calibration plots after recalibration of the models predicting referable diabetic retinopathy (EURODIAB grade ≥ 2) (A), sight-threatening diabetic retinopathy (EURODIAB grade ≥ 3) (B), and photocoagulated diabetic retinopathy (EURODIAB grade ≥ 4) (C) in the Diabetes Care System cohort. The plots depict the observed mean risk against the predicted mean retinopathy risk within quintiles of the predicted risk.

References

[1] Aspinall PA, Kinnear PR, Duncan LJ, Clarke BF (1983) Prediction of diabetic retinopathy from clinical variables and color vision data. Diabetes Care 6(2): 144-148. 10.2337/diacare.6.2.144
[2] Clarke PM, Gray AM, Briggs A, et al. (2004) A model to estimate the lifetime health outcomes of patients with type 2 diabetes: the United Kingdom Prospective Diabetes Study (UKPDS) Outcomes Model (UKPDS no. 68). Diabetologia 47(10): 1747-1759. 10.1007/s00125-004-1527-z
[3] Aspelund T, Thornorisdottir O, Olafsdottir E, et al. (2011) Individual risk assessment and information technology to optimise screening frequency for diabetic retinopathy. Diabetologia 54(10): 2525-2532. 10.1007/s00125-011-2257-7
[4] Semeraro F, Parrinello G, Cancarini A, et al. (2011) Predicting the risk of diabetic retinopathy in type 2 diabetic patients. J Diabetes Complications 25(5): 292-297. 10.1016/j.jdiacomp.2010.12.002
[5] Mehlsen J, Erlandsen M, Poulsen PL, Bek T (2012) Individualized optimization of the screening interval for diabetic retinopathy: a new model. Acta Ophthalmol 90(2): 109-114. 10.1111/j.1755-3768.2010.01882.x
[6] Tanaka S, Tanaka S, Iimuro S, et al. (2013) Predicting macro- and microvascular complications in type 2 diabetes: the Japan Diabetes Complications Study/the Japanese Elderly Diabetes Intervention Trial risk engine. Diabetes Care 36(5): 1193-1199. 10.2337/dc12-0958
[7] Scanlon PH, Aldington SJ, Leal J, et al. (2015) Development of a cost-effectiveness model for optimisation of the screening interval in diabetic retinopathy screening. Health Technol Assess 19(74): 1-116. 10.3310/hta19740
[8] Hippisley-Cox J, Coupland C (2015) Development and validation of risk prediction equations to estimate future risk of blindness and lower limb amputation in patients with diabetes: cohort study. BMJ 351: h5441. 10.1136/bmj.h5441
[9] Basu S, Sussman JB, Berkowitz SA, Hayward RA, Yudkin JS (2017) Development and validation of Risk Equations for Complications Of type 2 Diabetes (RECODe) using individual participant data from randomised trials. Lancet Diabetes Endocrinol 5(10): 788-798. 10.1016/S2213-8587(17)30221-8
[10] Dagliati A, Marini S, Sacchi L, et al. (2018) Machine Learning Methods to Predict Diabetes Complications. J Diabetes Sci Technol 12(2): 295-302. 10.1177/1932296817706375
[11] Garcia-Finana M, Hughes DM, Cheyne CP, et al. (2019) Personalized risk-based screening for diabetic retinopathy: A multivariate approach versus the use of stratification rules. Diabetes Obes Metab 21(3): 560-568. 10.1111/dom.13552
[12] Ochs A, McGurnaghan S, Black MW, et al. (2019) Use of personalised risk-based screening schedules to optimise workload and sojourn time in screening programmes for diabetic retinopathy: A retrospective cohort study. Plos Medicine 16(10). ARTN e100294510.1371/journal.pmed.1002945
